# Quantitative CT-based bone strength parameters for the prediction of novel spinal implant stability using resonance frequency analysis: a cadaveric study involving experimental micro-CT and clinical multislice CT

**DOI:** 10.1186/s41747-018-0080-3

**Published:** 2019-01-22

**Authors:** Daisuke Nakashima, Ken Ishii, Yuji Nishiwaki, Hiromasa Kawana, Masahiro Jinzaki, Morio Matsumoto, Masaya Nakamura, Takeo Nagura

**Affiliations:** 1 Department of Orthopedic surgery, Keio University School of Medicine, 35 Shinanomachi, Shinjuku, Tokyo Japan; 2 Department of Orthopedic surgery, International University of Health and Welfare School of Medicine, Narita, Chiba Japan; 3 Department of Environmental and Occupational Health, School of Medicine, Toho University, Tokyo, Japan; 4 Department of Dentistry and Oral Surgery, Keio University School of Medicine, Shinjuku, Tokyo Japan; 5 Department of Radiology, Keio University School of Medicine, Shinjuku, Tokyo Japan; 6 Department of Clinical Biomechanics, Keio University School of Medicine, Shinjuku, Tokyo Japan

**Keywords:** Bone density, Pedicle screws, Resonance frequency analysis, Torque, X-ray (microtomography)

## Abstract

**Background:**

To predict conventional test forces (peak torque and pull-out force) and a new test force (implant stability quotient [ISQ] value of a spinal pedicle screw) from computed tomography (CT) parameters, including micro-architectural parameters, using high-resolution micro-CT and clinical multislice CT (MSCT) in human cadaveric vertebrae.

**Methods:**

Micro-CT scans before/after screw insertion (*n* = 68) and MSCT scans before screw insertion (*n* = 58) of human cadaveric vertebrae were assessed for conventional test forces and ISQ value. Three-dimensional volume position adjustment between pre-insertion micro-CT and MSCT scans and post-insertion scans (micro-CT) was performed to extract the volume of the cancellous bone surrounding the pedicle screw. The following volume bone mineral density and micro-architectural parameters were calculated: bone volume fraction, bone surface density (bone surface/total volume (BS/TV)), trabecular thickness, trabecular separation, trabecular number, structure model index, and number of nodes (branch points) of the cancellous bone network/total volume (NNd/TV) using Spearman’s rank correlation coefficient with Bonferroni correction.

**Results:**

Conventional test forces showed the strongest correlation with BS/TV: peak torque, *ρ* = 0.811, *p* = 4.96 × 10^−17^(micro-CT) and *ρ* = 0.730, *p* = 7.87 × 10^−11^ (MSCT); pull-out force, *ρ* = 0.730, *p* = 1.64 × 10^−12^ (micro-CT) and *ρ* = 0.693, *p* = 1.64 × 10^−9^ (MSCT). ISQ value showed the strongest correlation with NNd/TV: *ρ* = 0.607, *p* = 4.01 × 10^−8^ (micro-CT) and *ρ* = 0.515, *p* = 3.52 × 10^−5^ (MSCT).

**Conclusions:**

Test forces, including the ISQ value, can be predicted using micro-CT and MSCT parameters. This is useful for establishing a preoperative fixation strength evaluation system.

## Key points

- Implant stability quotient (ISQ) value is a new test force that reflects *in vivo* stress.
- ISQ value and conventional test forces of spinal implant stability are different.
- These test forces could be predicted using both micro-CT and clinical multislice CT.
- Bone surface density is the most effective in predicting conventional test forces.
- Cancellous bone network/total volume branch points are suitable in predicting ISQ value.

## Background

Spinal instrumentation surgeries are popular, and an increasing number of surgeries is being performed annually [1, 2]. Implant stability is important in spinal instrumentation surgeries, as it ensures secure fixation of the implant to the bone and prevents implant fixation failure [3, 4]. In particular, in the field of spinal surgery, the rate of postoperative pedicle screw loosening has been reported to be up to 12% [5, 6]. Galbusera et al. [7] reported a screw loosening incidence of up to 60% among patients with osteoporosis. Pedicle screw loosening is one of the major indications for revision after spinal surgery [8]. An appropriate initial test force is important to achieve good surgical results and prevent screw loosening. To overcome these issues, both the prediction of test force using preoperative images and establishment of an intraoperative test force evaluation system are required.

With regard to the prediction of test force using preoperative images, some reports have demonstrated a correlation between the test force and bone mineral density (BMD) of the osteoporotic vertebra assessed using two-dimensional dual-energy x-ray absorptiometry (DEXA), which is one of the preoperative imaging modalities [9, 10]. Although computed tomography (CT) is also frequently used for planning spinal surgery, it is not yet commonly performed in clinical practice to predict the test force of a pedicle screw using imaging data.

The term *bone strength* indicates resistance to bone destruction and was defined by the National Institutes of Health Consensus 2000 Development Panel on Osteoporosis Prevention, Diagnosis, and Therapy [11]. BMD is frequently used as an indirect measure of bone strength and accounts for approximately 70% of bone strength. The remaining 30% of bone strength is considered to involve *bone quality*, which has been described as the flexibility and soundness of the bone structure. In CT, bone quality can be represented by digitising the three-dimensional (3D) structure of the bone with patterns such as honeycomb-, plate-, and beam-shaped patterns. These structures are represented by micro-architectural parameters [12]. Many recent studies have reported that BMD assessed using DEXA alone may not be sufficient to determine the strength of cancellous bone and that trabecular architecture may be an important predictor of bone strength and fracture risk [13, 14].

With regard to the establishment of an intraoperative test force evaluation system, pull-out force [3, 15, 16] and insertion torque [3, 17] are the test force measures generally used to evaluate screw stability. Pull-out force is measured destructively through laboratory testing and is defined as the maximum axial force required to pull a screw out from the bone [18]. Additionally, it is not possible to measure insertion torque more than once after screw fixation. Thus, pull-out force and insertion torque are not used as intraoperative measures.

We have previously reported [19] on a system involving resonance frequency analysis (RFA) [20–22] that can be used for the intraoperative evaluation of a pedicle screw (Osstell apparatus). With this system, the dedicated parameter implant stability quotient (ISQ) can be assessed, and its value ranges from 0 (lowest stability) to 100 (highest stability) [23]. An ISQ value of 0 represents a resonance frequency of approximately 3,000 Hz, and a value of 100 represents a resonance frequency of approximately 8,000 Hz [24]. This non-invasive and repeatable technique might indicate multidirectional test force [19, 23], which differs from insertion torque and pull-out force that reflect only axial force [19].

The present study aimed to predict conventional test forces (peak torque and pull-out force) and a new test force (ISQ value of a spinal pedicle screw) from CT parameters, including micro-architectural parameters, using experimental high-resolution micro-CT and clinical multislice CT (MSCT) in human cadaveric vertebrae.

## Methods

### Spinal screws

Monoaxial pedicle screws (Catalog no. CMS05135, Kyocera Medical Corporation, Kyoto, Japan) measuring 45 mm in length, with an outer threaded diameter of 5.5 mm, an inner threaded diameter of 3.8 mm (start point), and of 4.6 mm (end point), made of titanium alloy (Ti-6Al-4 V[ELI], American Society for Testing and Materials [ASTM] F136) were used in this study. To measure the ISQ value with the Osstell ISQ® system (Osstell Integration Diagnostics, Gothenburg, Sweden), two neodymium magnets (Magfine Corporation, Miyagi, Japan) were attached to the head of the pedicle screw as previously reported [19].

### Spinal dissection and treatment before the experiment

For the experimental use of fresh non-frozen human cadavers, written informed consent was obtained from each donor according to the ethical guidelines of our institution (approval number: 20070026). Dissection was performed as soon as possible post-mortem. All experiments were approved by the Ethics Committee (approval number: 20150385). Nine human thoracolumbar spinal sections (Th8–L5) were used in this study (mean donor age, 76 years; age range, 72–86 years; seven female and two male cadavers). The intervertebral disks and ligaments were dissected from the vertebrae. Vertebrae that showed spinal fusion (*n* = 4) or were damaged during preparation (*n* = 6) were excluded. The remaining vertebrae were visually inspected and diagnosed using micro-CT images by two spinal surgeons (11 and 27 years of experience), and vertebrae showing fractures or spinal metastases were excluded (*n* = 3). The specimens were individually wrapped in Ziploc polyethylene bags (S.C. Johnson & Son, Incorporated, Racine, WI) for micro-CT imaging [17].

### Micro-CT and MSCT imaging before pedicle screw insertion

After the scan of a BMD phantom (RATOC, Tokyo, Japan), the vertebrae were scanned using a micro-CT system (R_mCT2 FX, Rigaku Corporation, Tokyo, Japan) with the following settings: x-ray voltage, 90 kVp; tube current, 160 μA; exposure time, 120 s, continuous (non-stepping) rotation. For the BMD phantom and each vertebra, a stack of 400 cross-sectional slices, corresponding to a total height of 57 mm, was reconstructed with a slice-to-slice distance of 1 pixel (144 μm). The scan data of the BMD phantom were used to convert the CT number (Hounsfield units) to BMD (mg/cm^3^).

**Fig. 1:**
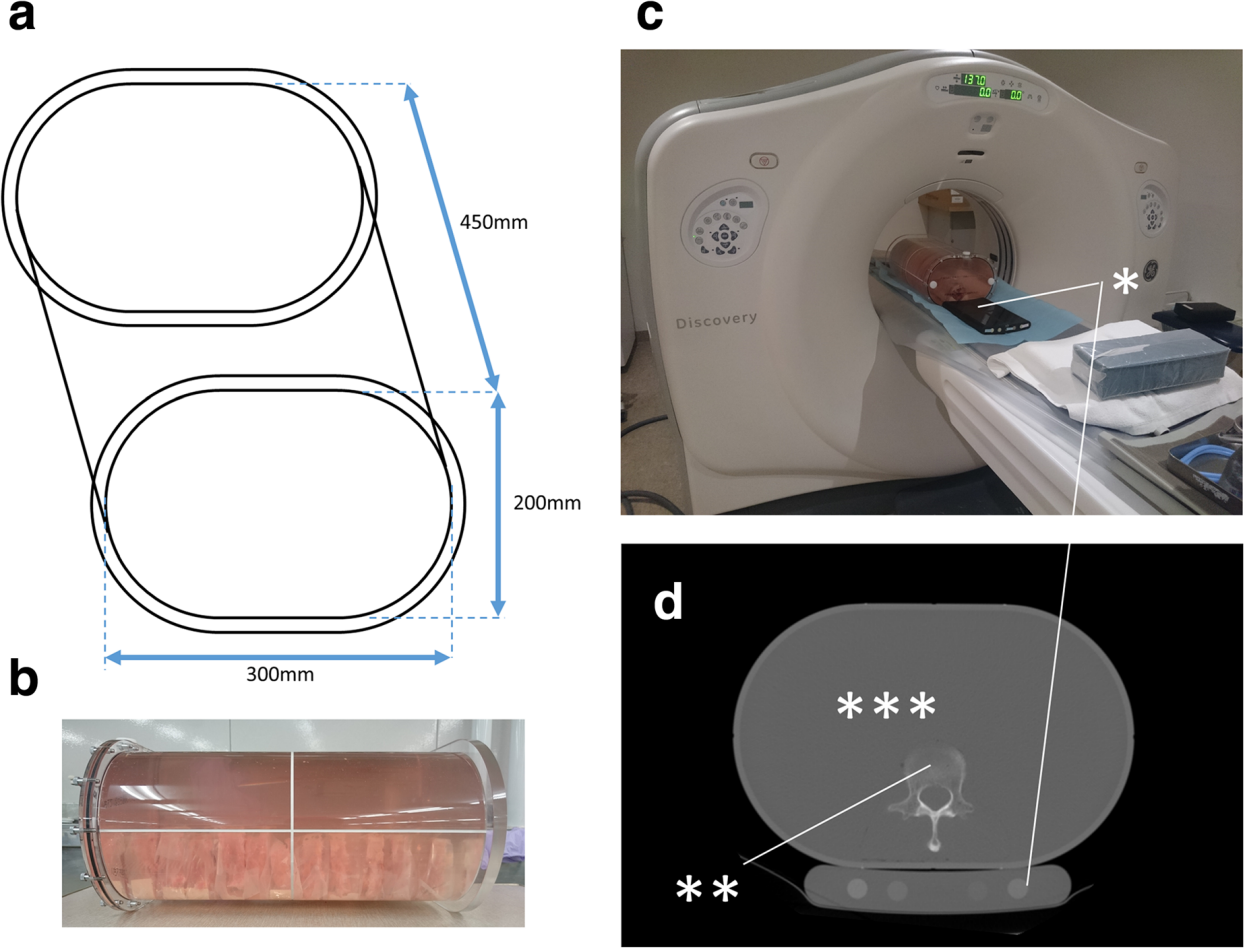
Multislice computed tomography. **a** Drawing of the in-house round rectangle-shaped tank. **b** Vertebrae in the tank. **c** Photograph of the tank and the phantom (*) in the scanner. **d** Axial image showing the phantom (*), one vertebra (**), and the surrounding water for mimicking a living body (***)

After micro-CT, MSCT was performed. The vertebrae were placed in an in-house rounded rectangle-shaped tank (Fig. 1a, b) that simulated the human body, and MSCT imaging was performed with a BMD phantom (B-MAS200, Fujirebio, Tokyo, Japan) (Fig. 1c, d) using an MSCT system (Discovery CT750, GE Healthcare, Chicago, IL) using a standard protocol (for the phantom, 120 kVp, 250 mA, voxel size 683 × 683 × 5,000 μm, field of view 350 × 350 mm; for the vertebra: 120 kVp, 250 mA, voxel size 293 × 293 × 625 μm, field of view 150 × 150 mm) (Fig. 1d). The scan data of the BMD phantom were used to convert the CT number to BMD (mg/cm^3^) [25].

### Fixation strength experiment

#### Pedicle screw insertion

Pedicle screw insertion was performed by a spinal surgeon. A pilot hole was drilled in the vertebra using an awl and a 20-mm probe without a tap. The pedicle screw was inserted 40 mm.

#### Insertion torque measurement

**Fig. 2:**
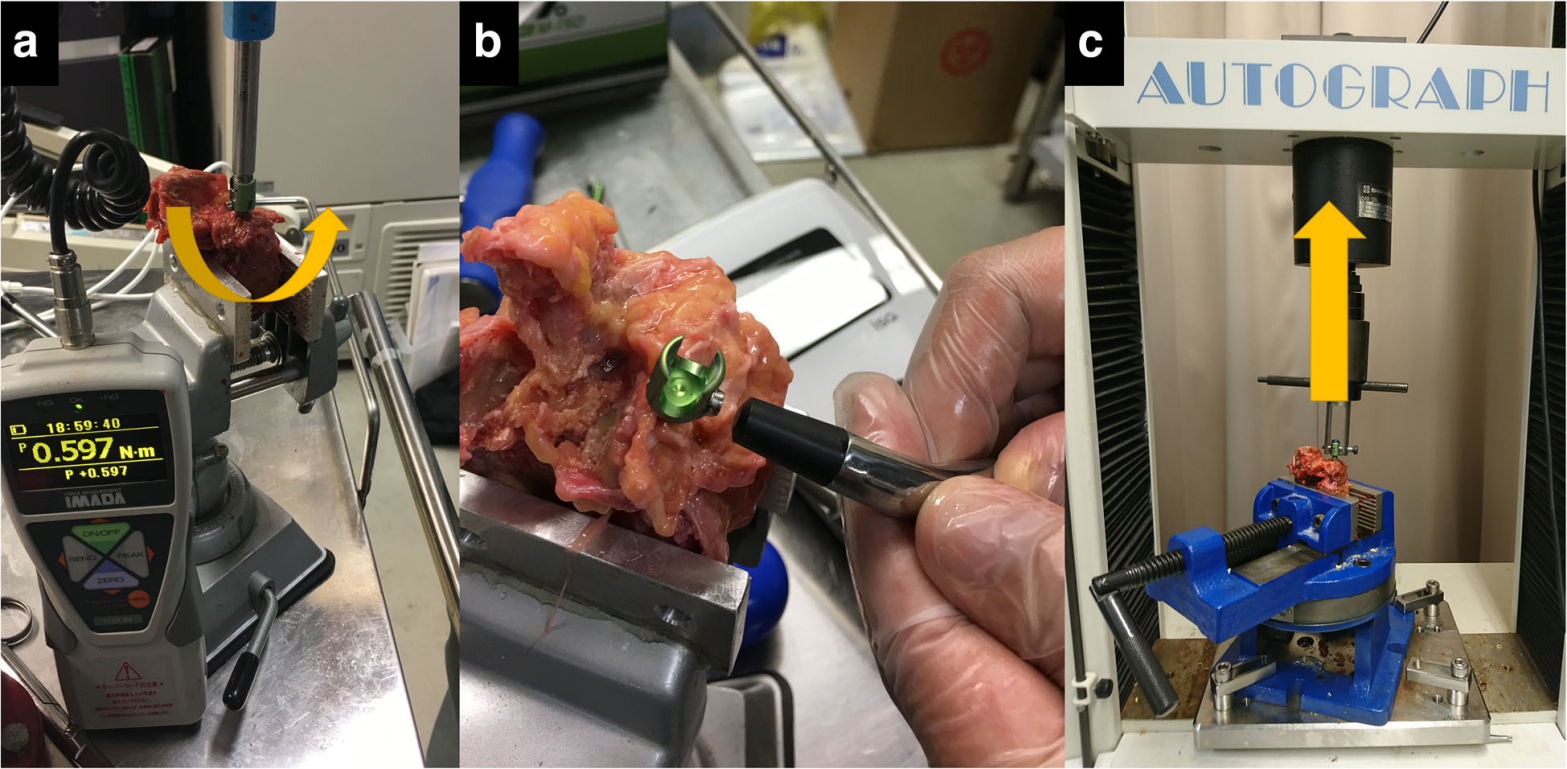
Test force measurement. **a** Peak torque measurement. **b** Implant stability quotient value measurement. **c** Pull-out force measurement

The digital torque gauge HTGA-5 N (Imada Company Limited, Aichi, Japan) was used to measure the insertion torque (peak torque [19, 26, 27]) at 40-mm insertion. The specifications of this torque gauge were as follows: accuracy ± 0.5% (full scale) ± 1 digit and sampling rate 2,000 data/s. The insertion torque (Newton meter, N m) is a moment force, and it increases progressively as the screw advances in the vertebra. This insertion torque peaks before the screw head comes in contact with the vertebra, which is defined as *peak torque*. This torque is experienced as the test force of the pedicle screw by the surgeon (Fig. 2a) [19, 26].

#### Resonance frequency analysis

It was conducted using a specific device (Osstell Integration Diagnostics, Gothenburg, Sweden) without screw contact after completion of the pedicle screw insertion as reported previously (Fig. 2b) [19]. Materials were not held by a fixture during measurement and were placed on a normal laboratory table instead. The pedicle screw was vibrated with a micromagnetic wave, which generated inertial forces owing to the mass of the magnets in a plane perpendicular to the axis of the screw.

#### Micro-CT imaging after pedicle screw insertion

**Fig. 3:**
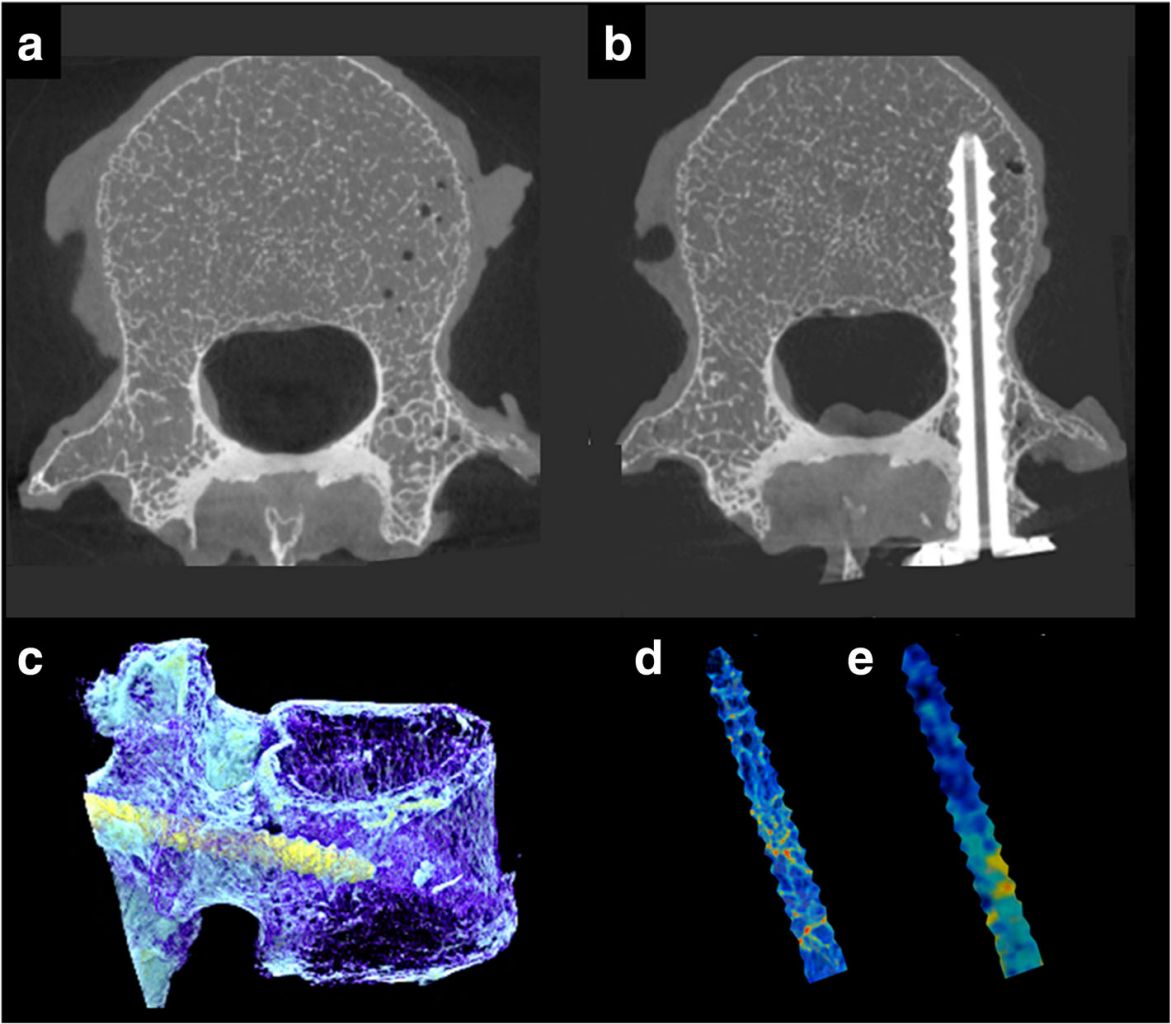
Micro-CT and MSCT imaging. **a** Micro-CT axial image of a vertebra. **b** Micro-CT axial image of a vertebra with a screw. **c** Three-dimensional micro-CT reconstruction of a vertebra with a screw. Cancellous bone area of the pedicle screw extracted by three-dimensional volume position adjustment between the pre- and post-insertion images: micro-CT (**d**) and MSCT (**e**)

Micro-CT imaging was performed again for the vertebrae with the pedicle screws using the same protocol as that before screw insertion (Fig. 3b).

#### Pull-out force measurement

The pull-out force measurement was performed according to ASTM-F543-07 testing standards [28]. The vertebrae were placed on a specially fabricated fixture with a self-position adjustment function to ensure vertical pull-out alignment. Then, they were held in the appropriate position on a base plate. The maximum pull-out force was measured using AG-IS 10 kN (Shimadzu Corporation, Kyoto, Japan; testing speed 5 mm/min) [28]. Strength was continuously recorded in 0.1-mm increments until its peak (Fig. 2c).

#### Volume BMD and micro-architectural parameters of the vertebrae

Imaging analysis was performed using dedicated software (TRI/3D-BON, RATOC, Tokyo, Japan) to calculate volume BMD and the micro-architectural parameters of the vertebrae. First, 3D volume position adjustment between pre-insertion (both micro-CT and MSCT) (Fig. 3a) and post-insertion (micro-CT) (Fig. 3b, c) images was performed, and the volume of the cancellous bone where the pedicle screw was present was extracted (Fig. 3d, e). The cutoff value was 120 mg/cm^3^. The cortex was extracted before calculating the 3D volume position adjustment. Volume BMD and micro-architectural parameters were calculated. The following micro-architectural parameters were assessed: bone volume fraction (bone volume/total volume: BV/TV, %), bone surface density (bone surface/total volume (BS/TV), mm^−1^), trabecular thickness (mm), trabecular separation (mm), trabecular number (mm^−1^), structure model index (SMI), and number of nodes per volume (NNd/TV, mm^−3^) [17, 29–31]. SMI is a measure for the relative number of rod- and plate-like trabecular bone structures, with values ranging from 0 (ideal plate-like structure) to 3 (ideal rod-like structure), values in between representing a mixture of plates and rods [32]. In recent years, SMI has frequently been used to investigate the relationship between CT and implant fixation strength in research [17, 32]. NNd/TV, unlike SMI, indicated the complexity of trabecular bone structure from the viewpoint of the number of bonding points of the trabecular beam structure.

### Statistical analysis

The three measures (peak torque, pull-out force, and ISQ value) and parameters of both micro-CT and MSCT were obtained. First, Spearman’s rank moment correlation coefficient (*ρ*) with Bonferroni correction was used to evaluate the relationship among the three measures. The significance level was set at *p* = 0.0166. Second, the same analysis was performed to evaluate the relationship between the three measures and the parameters of both micro-CT and MSCT. The significance level was set at *p* = 0.00625. All statistical analyses were performed using SPSS Statistics software version 24 (International Business Machines Corporation, Armonk, NY).

## Results

A total of 90 vertebrae were initially included. However, 13 vertebrae met the abovementioned exclusion criteria and 9 vertebrae were excluded due to pedicle destruction during screw insertion. Thus, 68 vertebrae were successfully examined with all assessments, except MSCT imaging parameter assessments. MSCT imaging parameter assessments could not be performed in 10 vertebrae owing to insufficient resolution. Therefore, 58 vertebrae were examined in the MSCT study. Table 1 summarises the three measures and the parameters of CT imaging.

**Table 1:** Summary of the three test forces and parameters of the vertebrae on micro-CT and MSCT

	Measure unit	Mean (standard deviation)
Test forces (*n* = 68)		
Peak torque	N m	678 (246)
Pull-out force	N	357 (130)
Implant stability quotient value	Arbitrary units	36.5 (7.90)
Micro-CT parameters (*n* = 68)		
Volume bone mineral density	mg/cm^3^	69.6 (30.5)
Bone volume/total volume	–	29.6 (10.7)
Bone surface/total volume	mm^−1^	1.80 (0.350)
Trabecular thickness	μm	328 (73.0)
Trabecular separation	μm	863 (328)
Trabecular number	mm^−1^	0.876 (0.170)
Structure model index	–	1.86 (0.534)
Node number/total volume	mm^−3^	0.647 (0.159)
Multislice CT parameters (*n* = 58)		
Volume bone mineral density	mg/cm^3^	183 (28.1)
Bone volume/total volume	–	91.1 (10.8)
Bone surface/total volume	mm^−1^	4.16 (0.903)
Trabecular thickness	μm	780 (252)
Trabecular separation	μm	81.0 (14.2)
Trabecular number	mm^−1^	1.28 (0.416)
Node number/total volume	mm^−3^	0.764 (0.0230)

*CT* computed tomography, *N* Newton

### Correlations among the three test forces

**Fig. 4:**
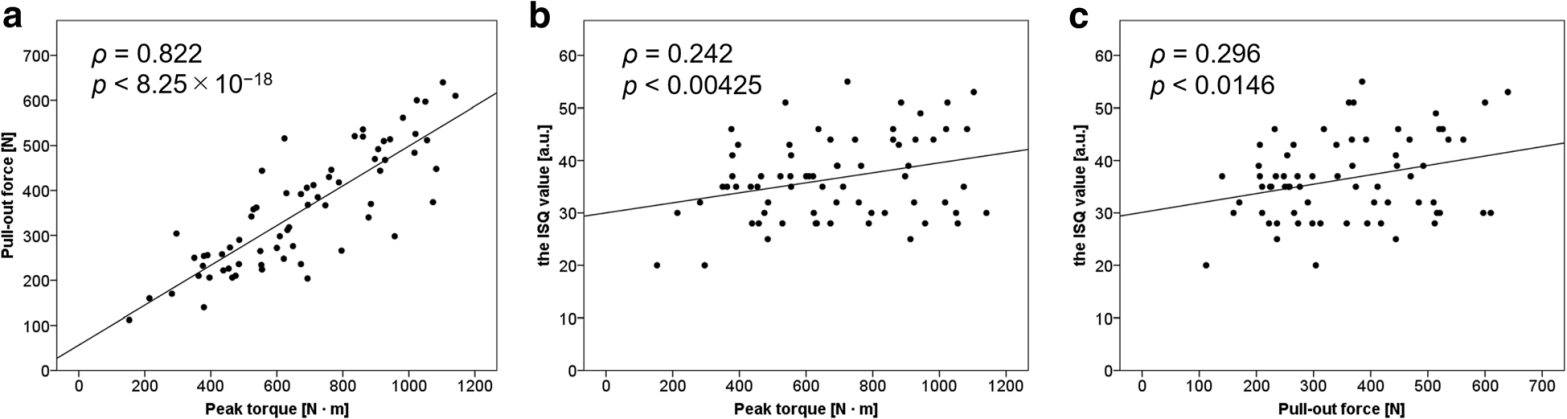
Scatter plots and best fit lines of linear regression among the three test forces. **a** Peak torque versus pull-out force. **b** Peak torque versus implant stability quotient (ISQ) value. **c** Pull-out force versus implant stability quotient value. *N*, Newton; *N m* Newton meter, *a.u.* arbitrary units

Peak torque showed a strong positive correlation with pull-out force (*ρ* = 0.822, *p* = 8.25 × 10^−18^) (Fig. 4a). The ISQ value showed weak positive correlations with peak torque (*ρ* = 0.242, *p* = 0.00425) (Fig. 4b) and pull-out force (*ρ* = 0.296, *p* = 0.0146) (Fig. 4c). These results are similar to those obtained in a model bone study [19].

### Micro-CT study: correlations between the three measures and imaging parameters

**Fig. 5:**
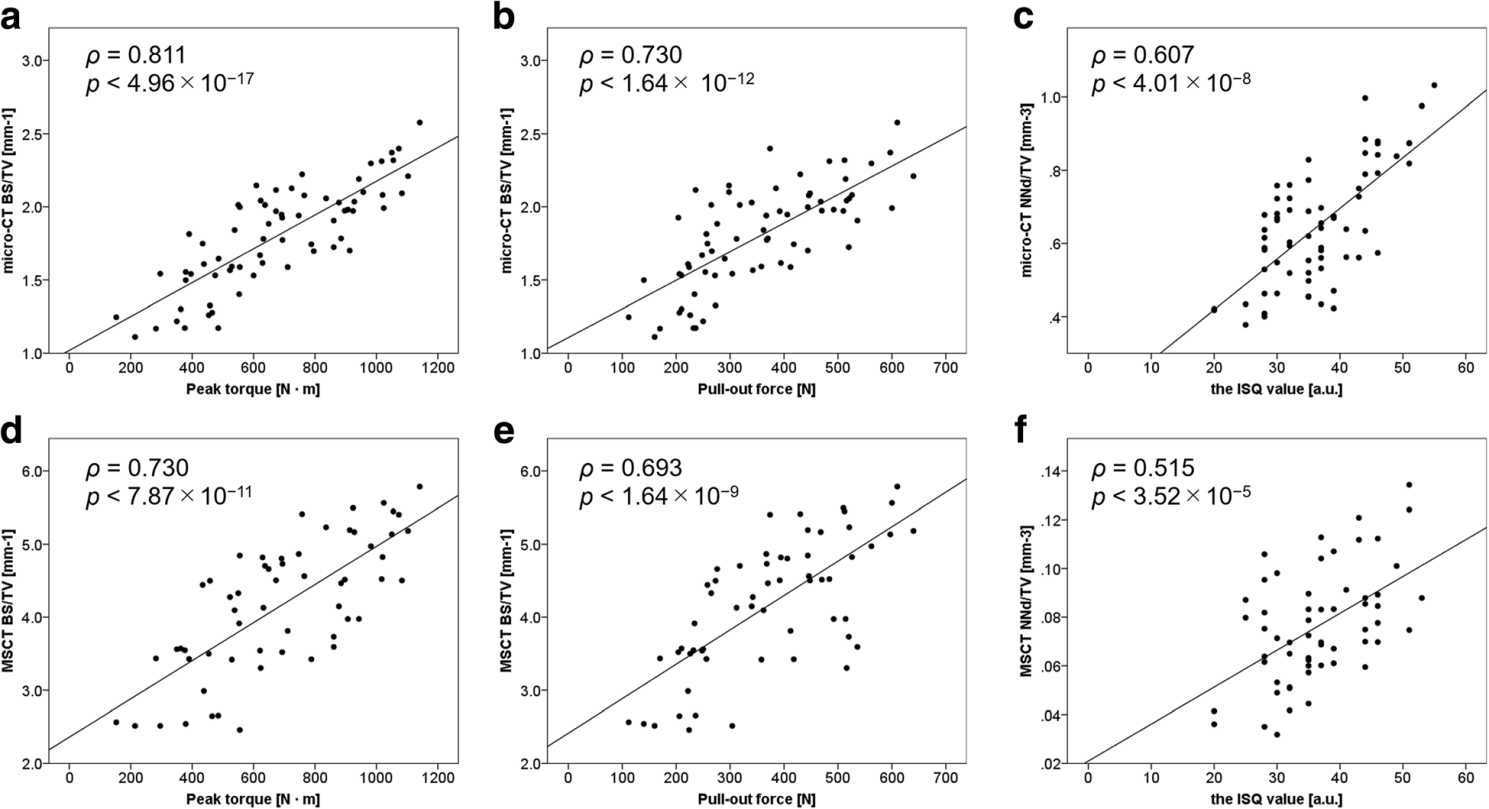
Scatter plots and best fit lines of linear regression between test forces and micro-architectural parameters (bone surface density) or node number (number of branch points of the cancellous bone network/total volume) on micro-CT and MSCT. **a** Peak torque versus bone surface density (bone surface [BS]/total volume [TV]) on micro-CT. **b** Pull-out force versus BS/TV on micro-CT. **c** Implant stability quotient (ISQ) value versus node number (NNd)/TV on micro-CT. **d** Peak torque versus BS/TV on MSCT. **e** Pull-out force versus BS/TV on MSCT. **f** ISQ value versus NNd/TV on MSCT. *N* Newton, *N m* Newton meter, *a.u.* arbitrary units

Table 2 summarises the correlations between the three test forces and CT imaging parameters. Both conventional test forces (peak torque and pull-out force) showed the strongest correlations with BS/TV (peak torque: *ρ* = 0.811, *p* = 4.96 × 10^−17^; pull-out force: *ρ* = 0.730, *p* = 1.64 × 10^−12^) (Fig. 5a, b). Additionally, both test forces showed lower but significant correlations with volume BMD and micro-architectural parameters, except BS/TV (*ρ* ranging from − 0.588 to 0.582). Interestingly, the ISQ value showed the strongest correlation with NNd/TV (*ρ* = 0.607, *p* = 4.01 × 10^−8^), unlike the conventional test forces (Fig. 5c). However, it was not significantly correlated with volume BMD, BV/TV, BS/TV, trabecular thickness, trabecular separation, trabecular number, and SMI.

**Table 2:** Correlation coefficients and *p* values for the three test forces versus CT parameters on micro-CT and multislice CT

		Micro-CT (*n* = 68)		Multislice CT (*n* = 58)	
		*ρ*	*p*	*ρ*	*p*
Peak torque versus	Volume bone mineral density	0.520	5.46 × 10^−6^*	0.463	2.50 × 10^−4^*
	Bone volume/total volume	0.550	1.19 × 10^−6^*	0.334	0.0103
	Bone surface/total volume	0.811	4.96 × 10^−17^*	0.730	7.87 × 10^−11^*
	Trabecular thickness	0.427	2.79 × 10^−4^*	0.479	1.41 × 10^−4^*
	Trabecular separation	− 0.588	1.38 × 10^−7^*	− 0.267	0.0424
	Trabecular number	0.582	1.95 × 10^−7^*	− 0.489	9.66 × 10^−5^*
	Structure model index	− 0.437	1.96 × 10^−4^*	N.A.	N.A.
	Node number/total volume	0.428	2.71 × 10^−4^*	− 0.069	0.608
Pull-out versus	Volume bone mineral density	0.453	1.06 × 10^−4^*	0.414	0.00124*
	Bone volume/total volume	0.467	5.86 × 10^−5^*	0.320	0.0143
	Bone surface/total volume	0.730	1.64 × 10^−12^*	0.693	1.64 × 10^−9^*
	Trabecular thickness	0.361	0.00250*	0.416	0.00118*
	Trabecular separation	− 0.519	5.68 × 10^−6^*	− 0.272	0.0391
	Trabecular number	0.529	3.54 × 10^−6^*	− 0.411	0.00137*
	Structure model index	− 0.360	0.00253*	N.A.	N.A.
	Node number/total volume	0.483	3.02 × 10^−5^*	− 0.108	0.421
Implant stability quotient value versus	Volume bone mineral density	0.0722	0.558	− 0.103	0.444
	Bone volume/total volume	0.0666	0.589	− 0.0760	0.571
	Bone surface/total volume	0.237	0.0521	0.201	0.130
	Trabecular thickness	− 0.0542	0.661	− 0.106	0.427
	Trabecular separation	− 0.158	0.197	0.0770	0.566
	Trabecular number	0.264	0.0299	0.0830	0.536
	Structure model index	− 0.0134	0.914	N.A.	N.A.
	Node number/total volume	0.607	4.01 × 10^−8^*	0.515	3.52 × 10^−5^*

*CT* computed tomography, *N*.*A*. not available

*Signifiant *p* value

### MSCT study: correlations between the three measures and imaging parameters

Both conventional test forces (peak torque and pull-out force) showed the strongest correlations with BS/TV (peak torque: *ρ* = 0.730, *p* = 7.87 × 10^−11^; pull-out force: *ρ* = 0.693 *p* = 1.64 × 10^−9^) (Fig. 5d, e). Additionally, both test forces showed lower but significant correlations with volume BMD, trabecular thickness, and trabecular number (*ρ* ranging from − 0.489 to 0.479). The ISQ value showed the strongest moderate correlation with NNd/TV (*ρ* = 0.515, *p* = 3.52 × 10^−5^), similar to the finding in the micro-CT study (Fig. 5f). The ISQ value showed a lower but significant correlation with BS/TV (*ρ* = 0.267, *p* = 0.0427). However, it was not significantly correlated with volume BMD, BV/TV, BS/TV, trabecular thickness, trabecular separation, and trabecular number. SMI could not be calculated because of the lower resolution of MSCT images compared with that of micro-CT images.

## Discussion

The present study found that peak torque and pull-out force had the strongest correlations with BS/TV and that the ISQ value had the strongest correlation with NNd/TV on micro-CT and MSCT. Our findings indicate that test forces, including the ISQ value, can be predicted by CT parameters on both micro-CT and MSCT. Many trials have evaluated the stability of orthopedic implants (i.e., insertion torque and pull-out force) [9, 15, 17, 33–35]. To our knowledge, this is the first study to predict these test forces with micro-architectural parameters in the field of spinal surgery.

A strong relationship was found between peak torque and pull-out force (Fig. 4a), suggesting that peak torque, which is close to the operator’s hand, may be similar to pull-out force, which reflects axial load [18]. Siddiqui et al. [36] reported a strong correlation between the subjective assessment of screw hold and pull-out force. A similar result was obtained in the present study. In this study, RFA was used to evaluate pedicle screw stability. RFA involving the Osstell ISQ system has been used to evaluate dental implant stability [23]. Our research group [19] reported that RFA in spinal surgery reflects multidirectional load, which represents *in vivo* physiological loading conditions unlike conventional test forces (insertion torque and pull-out force), in a model bone study. In the present study using cadaveric vertebrae, the correlation of RFA with conventional test forces was low (Fig. 4b, c), similar to the finding in the previous report [19].

With regard to volume BMD and micro-architectural parameters on micro-CT, conventional test forces showed significant correlations with all parameters (Table 2). Particularly, BS/TV showed the strongest correlations with peak torque and pull-out force (Fig. 5a, b). The micro-architectural parameters used were microscopic measurements of the cancellous bone structure mainly obtained from micro-CT, and they are currently used in preclinical research [4, 17, 37, 38]. Variations in micro-architectural parameters have been reported to affect the stability of an implant according to laboratory failure testing (push-in force, pull-out force, and plateau torque) [17, 37–39].

The significant correlations of volume BMD, BV/TV, and SMI with the two conventional test forces observed in this study are in agreement with those reported in previous studies using a cadaveric femoral head [37, 38]. SMI is a measure of the relative number of rod- and plate-like trabecular structures, with values ranging from 0 (plate-like structure) to 3 (rod-like structure), with values between 0 and 3 representing a mixture of plates and rods [31, 32, 40]. These results indicate that a plate-like bone structure with high BMD or BV exhibits high test forces, as reported in a previous study [17]. Among the volume BMD and micro-architectural parameters in this study, BS/TV was the parameter that best predicted the two test forces by itself. The BS/TV value rises when the bone quality improves with high BMD.

On the other hand, the aforementioned parameters did not show similar regression coefficients for the ISQ value. NNd/TV, which is the number of branch points of the cancellous bone network/total volume of the measurement region [29], showed the strongest regression coefficient for the ISQ value (Fig. 5c). NNd/TV is the parameter mainly used for node-strut analysis [29, 41] to evaluate trabecular connectivity. NNd/TV represents the complexity of bone structure (the characteristic of the beam structure, i.e., bone quality), whereas BS/TV represents the bone surface area that is close to BMD. Generally, this parameter is used to evaluate the effects of drugs on osteoporosis [29, 30]. The finding indicates that bone quality (trabecular connectivity) has the best correlation with the ISQ value. The correlation coefficients between conventional test forces and BS/TV and that between the ISQ value and NNd/TV were lower but sufficient in the MSCT study when compared to the findings in the micro-CT study (Fig. 5d–f).

In recent years, micro-architectural parameters from MSCT imaging have been reported [42]. In many MSCT studies, cadaveric investigations were performed using MSCT with all soft tissues removed and exposed to air [43]. Under such a condition, no surrounding tissue that can affect MSCT imaging is present, and this imaging condition is much better than the clinical imaging condition of a living body. In order to replicate the situation noted in a living human, we placed the vertebrae in a tank simulating the human body and performed MSCT imaging (Fig. 1a–d). As sufficient correlation was obtained even with examination under strict and disadvantageous circumstances in this study, we believe that the prediction of test forces from preclinical images is sufficiently possible even with the clinical MSCT images. In the future, the correlation coefficients could be improved through the development of high-resolution clinical CT. The present study has some limitations. First, we did not consider the influence of the firmness of the cortical bone of the pedicle itself on the test forces. Currently, a device capable of substantially and non-invasively measuring the bone strength itself is absent. Second, micro-CT imaging was performed on excised specimens in an *in vitro* setting. Therefore, it did not account for the variability of tissues surrounding the bones among the different cadavers. Third, although the specimens were placed in a simulated body, MSCT was performed in an *in vitro* setting. Fourth, we did not perform a DEXA evaluation of BMD, which remains the most used tool in clinical practice. Therefore, a correlation between DEXA parameters (e.g., BMD, trabecular bone score) might be of further interest. Fifth, the vertebrae with screws were not scanned using MSCT. Although micro-CT alone is sufficient for screw position confirmation, it is more desirable to use the same resolution image for the 3D volume position adjustment. Nevertheless, the results of this study will be useful in clinical research with the same equipment in the future.

In conclusion, we showed that both micro-CT and MSCT parameters affect the test forces of the pedicle screw (not only conventional test forces but also the ISQ value). Therefore, if their values are evaluated preoperatively, they can be used in the prediction of initial test forces and in preoperative planning (e.g., fixed vertebrae number and implant selection).

## Abbreviations

3D: Three-dimensional
ASTM: American Society for Testing and Materials
BMD: Bone mineral density
BS: Bone surface
BV: Bone volume
CT: Computed tomography
DEXA: Dual-energy x-ray absorptiometry
ISQ: Implant stability quotient
RFA: Resonance frequency analysis
SMI: Structure model index
TV: Total volume
